# Ferrite-Core Four-Quadrant Rotating Eddy Current Probe for Enhanced Subsurface Defect Detection of Any Orientation

**DOI:** 10.3390/s26206495

**Published:** 2026-10-14

**Authors:** Alessandro Sardellitti, Federico Carere, Angelo D’Aguanno, Silvia Sangiovanni, Marco Laracca

**Affiliations:** 1 Department of Engineering and Science, Universitas Mercatorum, 00186 Rome, Italy; alessandro.sardellitti@unimercatorum.it; 2 Department of Information Science and Technology, Telematic University Pegaso, 80132 Naples, Italy; 3 Department of Electrical and Energy Engineering, Sapienza University of Rome, 00184 Rome, Italy; angelo.daguanno@uniroma1.it (A.D.); silvia.sangiovanni@uniroma1.it (S.S.); marco.laracca@uniroma1.it (M.L.)

**Keywords:** eddy current testing, rotating eddy current, ferrite core, non-destructive testing, subsurface defect detection, finite element analysis, magnetic sensors

## Abstract

Deep subsurface defect detection (due to the so-called skin effect) and sensitivity in the detection of any orientation defects remain two of the main challenges in Eddy Current Testing (ECT). Although low-frequency excitation increases the penetration depth of the induced eddy currents, it also reduces the intensity of the generated magnetic field, significantly degrading the inspection capability. Rotating eddy current probes overcome the dependence on defect orientation, but their performance remains limited for deep subsurface defects. This paper proposes a ferrite-core four-quadrant probe that combines orientation-independent inspection with magnetic flux concentration provided by ferrite cores integrated inside the excitation coils. The proposed probe is first investigated through finite element analysis and then experimentally validated through a direct comparison with the corresponding air-core configuration. Both numerical and experimental results demonstrate that the ferrite-core configuration systematically enhances the inspection capability, with improvements ranging from approximately 22% for shallow defects to over 100% for the deepest investigated ones. Moreover, the proposed probe successfully detects defects buried up to 6 mm below the inspected surface, whereas the corresponding air-core configuration fails under the same operating conditions. These results demonstrate the potential of ferrite-core integration for extending the applicability of REC probes to deep subsurface inspections.

## 1. Introduction

Structural components employed in industrial plants, civil infrastructures, aerospace systems, railway transportation, and oil and gas facilities are continuously exposed to degradation mechanisms such as fatigue, corrosion, and crack propagation. Early detection and characterization of these defects are essential to ensure structural integrity, operational safety, and effective maintenance planning [1,2]. In this context, Non-Destructive Testing (NDT) techniques enable the inspection of structural components without affecting their functional or mechanical properties, providing reliable information on their integrity and degradation state [3,4].

Among the available NDT techniques, Eddy Current Testing (ECT) is one of the most widely adopted methods for inspecting conductive materials owing to its non-contact operation, high inspection speed, ease of automation, and high sensitivity to material discontinuities. These characteristics have promoted its application in crack detection, corrosion assessment, thickness measurement, and material characterization across a broad range of industrial sectors [5,6,7,8,9,10,11].

Despite these advantages, the inspection capability of conventional ECT is fundamentally limited by two main challenges: the skin effect and the defect orientation with respect to the eddy current path.

Regarding the skin effect, increasing the excitation frequency strengthens the induced magnetic field but confines the eddy currents close to the specimen surface. Conversely, reducing the excitation frequency increases the penetration depth while significantly decreasing the magnetic field intensity and, consequently, the signal-to-noise ratio (SNR) [12]. As a result, reliable detection of deep subsurface defects remains one of the major challenges in eddy current inspection [13,14,15]. Several approaches have been proposed to overcome this limitation.

Early studies mainly focused on pulsed eddy current testing (PECT) and multi-frequency ECT combined with advanced signal processing techniques to improve defect detectability under low-SNR conditions [7,9,13,14,16,17]. The use of these excitation signals exploits a broad spectrum to analyze the specimen under test at different penetration depths. These approaches provide richer information than conventional single-frequency ECT and are particularly attractive for subsurface defect characterization. However, the response associated with the low-frequency components is generally weak and may require highly sensitive magnetic sensors and/or advanced signal-processing techniques to achieve an adequate SNR [8,13,14,18].

A complementary approach consists of modifying the magnetic circuit of the probe. Ferrite-core, pot-core, E-core and metamaterial-based configurations can concentrate the excitation flux toward the analyzed specimen, reduce magnetic leakage into the surrounding air, and increase the induced eddy-current density [8,9,15,18,19,20]. These effects are especially beneficial under low-frequency excitation, where improved penetration is otherwise accompanied by reduced sensitivity. For instance, probes with ferrite structures have demonstrated subsurface defect analysis several millimeters below the inspection surface [8,9,15,18,19,20]. Nevertheless, these probe architectures are mainly aimed at improving magnetic coupling, penetration capability, and SNR, and do not inherently remove the dependence of the ECT response on crack orientation.

Regarding the defect orientation with respect to the eddy current path, a reduction in the defect detection sensitivity can be observed, particularly for defects characterized by one dimension that is negligible with respect to the others, such as cracks. In these cases, maximum sensitivity is achieved when the induced eddy current paths intersect the crack approximately orthogonally, whereas defects aligned with the current flow generate much weaker electromagnetic perturbations [21,22,23,24]. Rotating Eddy Current (REC) techniques overcome this limitation by generating rotating eddy current distributions through multiple excitation coils, enabling orientation-independent inspection [21,22].

Ferrite cores have been integrated into REC probes to improve the SNR ratio while preserving the orientation-independent inspection capability. Trung et al. demonstrated, both numerically and experimentally, that ferrite cores placed inside the excitation coils increase the magnetic flux concentration and the measured signal [23,25]. However, these investigations were mainly focused on superficial cracks and were experimentally carried out at excitation frequencies around 10 kHz. Therefore, although they demonstrate the benefit of ferrite integration in REC techniques, they do not specifically investigate the low-frequency operating regime required for the detection of deep subsurface defects, where the reduction in magnetic field intensity and SNR becomes particularly critical.

The present work addresses this specific gap by investigating the integration of ferrite cores into a Four-Quadrant REC probe under low-frequency operating conditions selected specifically for deep subsurface inspection [24]. The proposed approach therefore combines two complementary strategies: the orientation-independent capability of rotating eddy currents and the magnetic-flux concentration provided by ferrite cores. The aim is to assess whether ferrite cores introduction can compensate for the loss of sensitivity associated with low-frequency REC inspection while preserving the capability of detecting defects regardless of their orientation. This proposal is evaluated through finite-element analysis and a direct experimental comparison with the corresponding air-core configuration.

The remainder of the paper is organized as follows. Section 2 introduces the operating principle of the proposed probe together with the adopted finite element model and the corresponding numerical investigation. Section 3 describes the experimental set-up and the analyzed case studies. Section 4 presents and discusses the experimental validation results. Finally, Section 5 summarizes the main conclusions of the paper and outlines possible future developments.

## 2. Theoretical Framework and Numerical Analysis

### 2.1. Theoretical Framework

This section briefly summarizes the operating principle of the previously developed REC technique and introduces the probe configuration adopted throughout this work.

**Figure 1:**
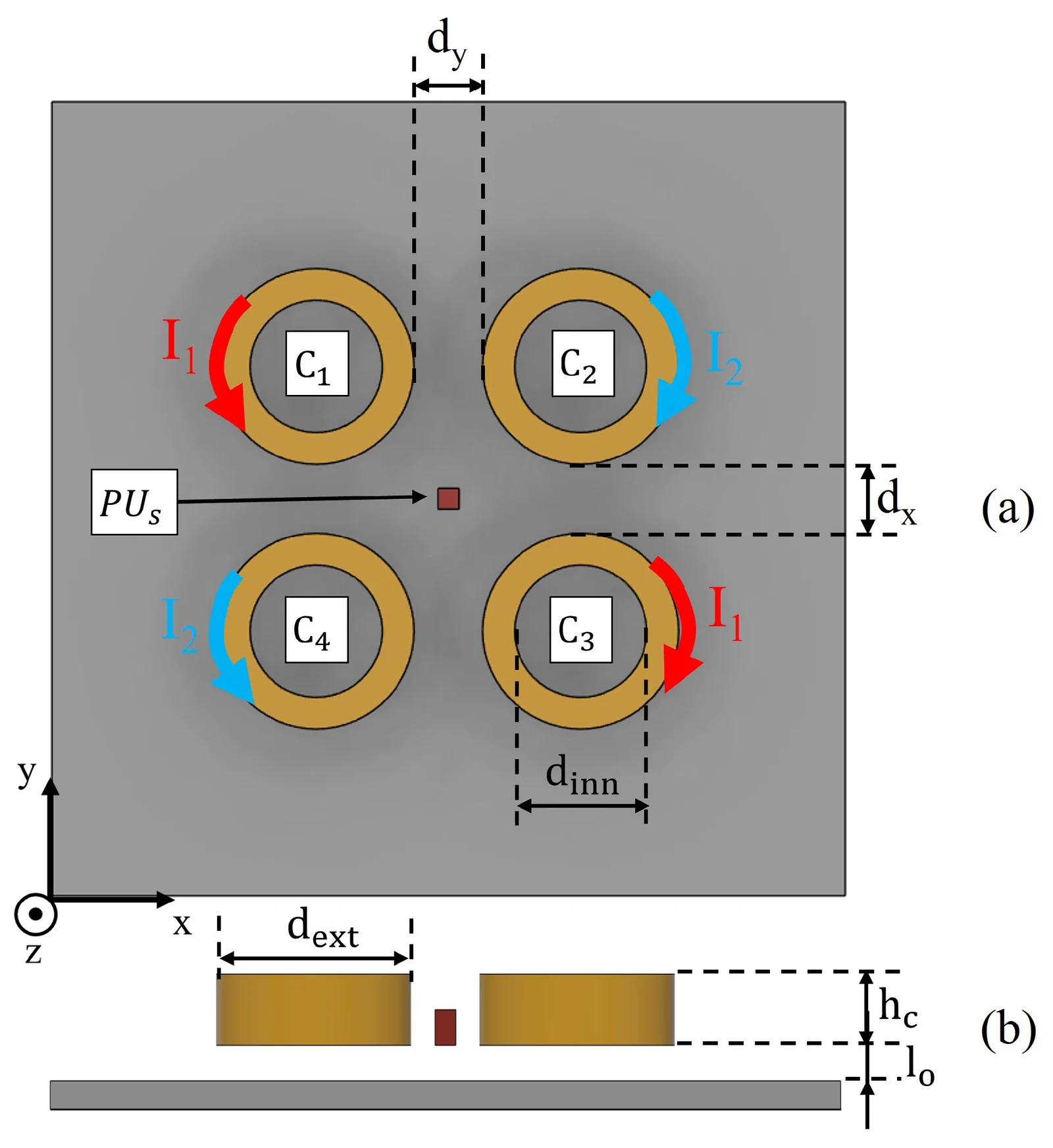
Geometry of the proposed ferrite-core Four-Quadrant (4Q) probe: (**a**) top view showing the four excitation coils and the central pick-up sensor; (**b**) side view highlighting the main geometrical parameters.

The adopted probe consists of four identical excitation coils symmetrically arranged around a central pick-up magnetic sensor ($PU_{s}$), which measures the z-component ($B_{z}$) of the magnetic field, as shown in Figure 1. The excitation system is organized into two orthogonal channels, namely coils $C_{1}$–$C_{3}$ and coils $C_{2}$–$C_{4}$, supplied by the excitation currents $I_{1}$ and $I_{2}$, respectively. The main geometrical parameters defining the probe are the external and internal coil diameters ($d_{ext}$ and $d_{inn}$), the distances between adjacent coils ($d_{x}$ and $d_{y}$), the excitation coil height ($h_{c}$), and the lift-off ($l_{0}$), defined as the distance between the probe and the specimen surface.

The operating principle of the probe relies on the generation of two orthogonal eddy current distributions inside the conductive specimen. The first excitation channel (i.e., $C_{1}$–$C_{3}$) generates a first eddy current path, whereas the second channel (i.e., $C_{2}$–$C_{4}$) generates a second orthogonal distribution. The superposition of these two contributions produces the resultant eddy current path, whose orientation is controlled by properly adjusting the amplitudes of the excitation currents.

Considering $I_{b}$ as the base excitation current and θ as the desired orientation of the resultant eddy current distribution, the two excitation currents are defined as

(1) $$I_{1}=I_{b}\left(\cos\theta +\sin\theta \right),$$

(2) $$I_{2}=I_{b}\left(-\cos\theta +\sin\theta \right).$$

According to (1) and (2), the amplitudes of the two excitation currents determine the orientation of the induced eddy current distribution while preserving its overall magnitude. Consequently, all the investigated orientations are generated under equivalent excitation conditions, ensuring comparable inspection sensitivity independently of the selected eddy current direction [24].

**Figure 2:**
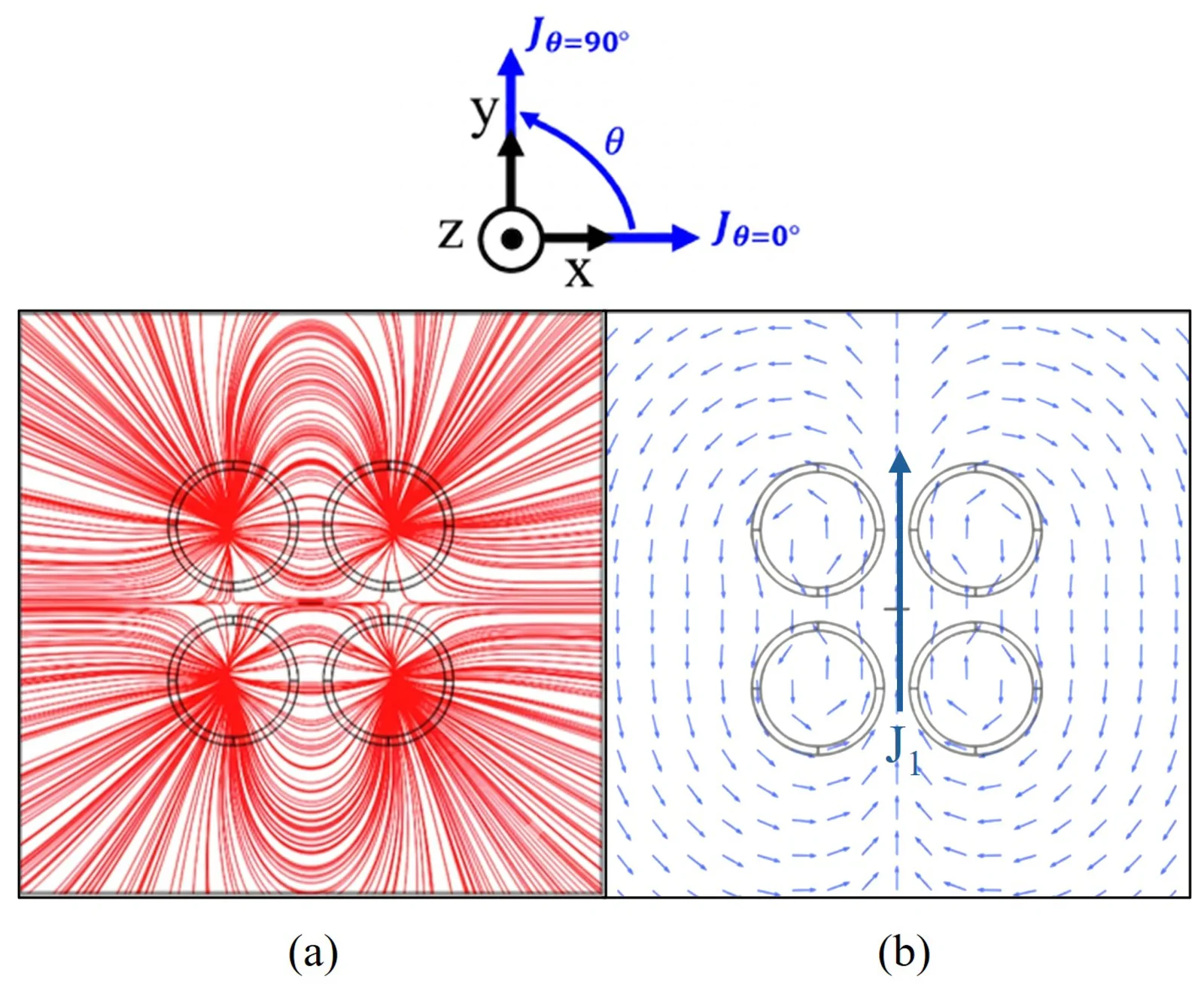
Electromagnetic behavior of the proposed REC excitation strategy for $\theta =90^{\circ }$: (**a**) magnetic flux distribution generated by the excitation system; (**b**) induced eddy current distribution inside the conductive specimen.

The electromagnetic behavior associated with the proposed excitation strategy is illustrated in Figure 2. Figure 2a reports the magnetic flux distribution obtained for $\theta =90^{\circ }$, corresponding to the operating condition $I_{1}=I_{2}$. The associated eddy current distribution induced inside the conductive specimen is shown in Figure 2b, where the resultant current density is mainly oriented along the positive *y* direction.

Based on this operating principle, the following subsection introduces the finite element model adopted to investigate the electromagnetic behavior of the proposed ferrite-core probe and to directly compare its performance with the corresponding air-core configuration.

### 2.2. Finite Element Analysis

The proposed ferrite-core probe was preliminarily investigated through finite element analysis carried out in COMSOL Multiphysics 6.0 using the “Magnetic Fields” interface and considering a frequency-domain analysis. The numerical model was developed to assess the influence of the ferrite cores on the electromagnetic response of the probe through a direct comparison with the corresponding air-core configuration.

The computational domain includes the excitation probe, the conductive specimen, and the surrounding air region. The excitation coils were implemented using the “Multi-Turn Coil” feature, whereas the ferrite cores were modeled as isotropic linear magnetic materials with negligible electrical conductivity. Although the relative permeability of the material composing the Mn-Zn ferrite powder is provided by the manufacturer, the effective permeability of the cores depends on the packing of the ferrite powder (i.e., ferrite grain size) and the resulting ferrite–air composition within the volume of the coil support. Therefore, the effective relative permeability was determined through model fitting by comparing the magnetic field obtained from the numerical model with that experimentally measured using the ferrite-core probe on a defect-free specimen under the same operating conditions. Applying this approach, the effective relative permeability $\mu_{r}=5$ provided the best fitting between the experimental and numerical magnetic fields and is then used in the finite element analysis reported in this paper.

The entire domain was discretized using the Physics-Controlled Mesh available in COMSOL, adjusting the mesh size for the different components of the model to accurately reproduce both the magnetic flux distribution and the induced eddy current paths.

The geometrical characteristics of the proposed probe are summarized in Table 1. The same probe geometry was adopted throughout both the numerical and experimental investigations in order to isolate the contribution of the ferrite cores from any geometrical effect.

Two aluminum specimens were considered to investigate the probe performance under different inspection conditions. Both specimens have planar dimensions of 200×200 mm^2^ and differ only in thickness, equal to 4 mm and 10 mm, respectively. The material was modeled as aluminum alloy AW6082 with electrical conductivity equal to 27.2 MS/m and relative magnetic permeability equal to 1.

**Figure 3:**
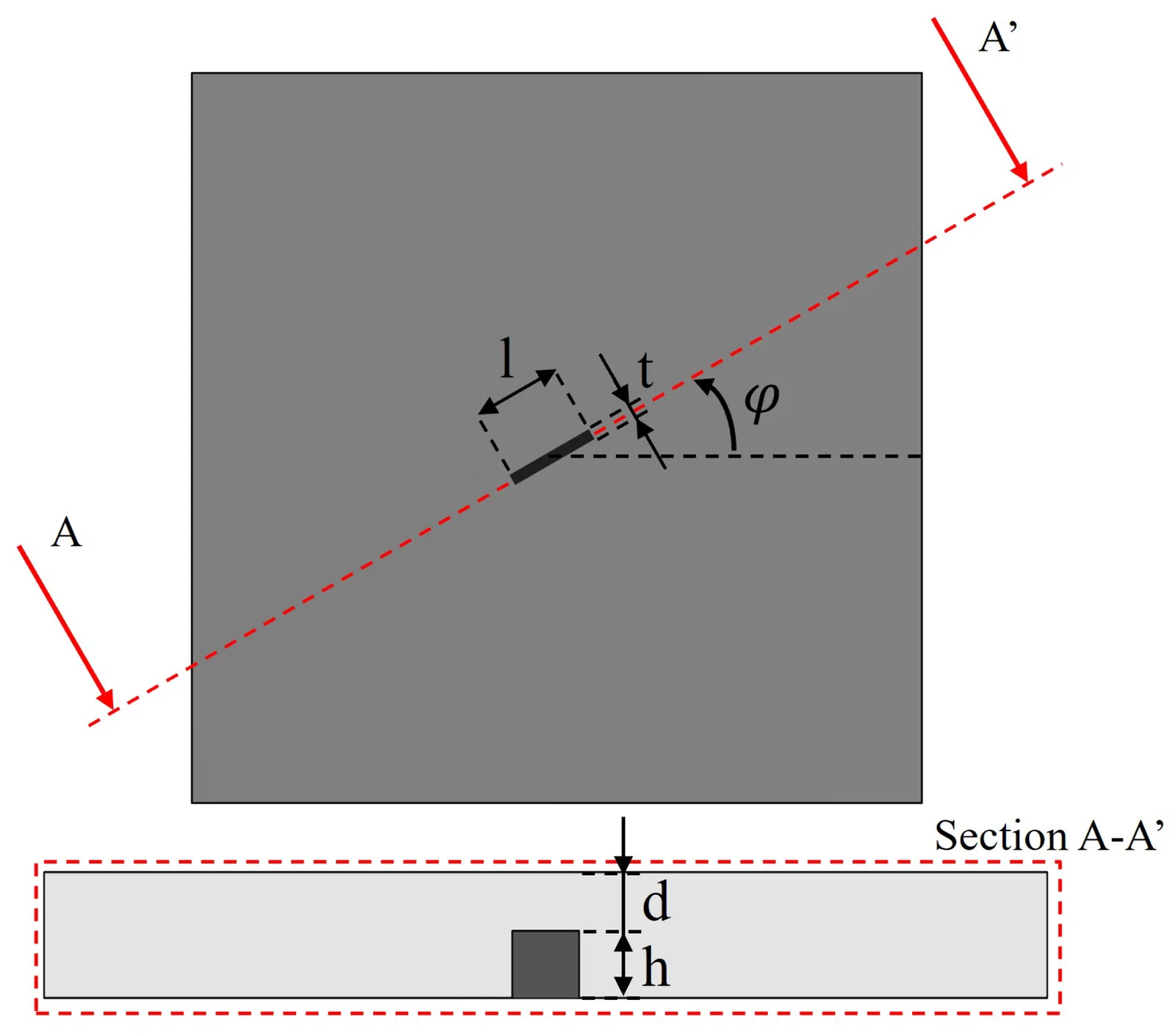
Definition of the geometrical parameters adopted to describe the investigated subsurface defects.

The investigated defects were modeled as air-filled rectangular cracks characterized by five geometrical parameters, namely the crack length (*l*), thickness (*t*), height (*h*), depth (*d*), and orientation angle (φ), as illustrated in Figure 3. The depth is defined as the distance between the inspected surface and the upper surface of the crack, whereas the crack height is measured along the specimen thickness. The complete set of analyzed defects is summarized in Table 2.

The selected configurations cover progressively more challenging inspection scenarios by increasing the defect depth for each specimen thickness.

Regarding the electrical excitation parameters, the base excitation current was set to $I_{b}=300$ mA, while $I_{1}$ and $I_{2}$ were varied according to (1) and (2) to rotate the resultant eddy current distribution with an angular resolution of $5^{\circ }$.

The excitation frequency was selected according to the specimen thickness and the depth of the investigated defects, taking into account the skin effect. In particular, lower excitation frequencies increase the skin depth and therefore promote a deeper penetration of the induced eddy currents, although at the expense of a reduced magnetic field intensity. Accordingly, an excitation frequency of 500 Hz was adopted for the 4 mm-thick specimen, whereas a lower frequency of 200 Hz was selected for the 10 mm-thick specimen, corresponding to skin depths of 4.32 mm and 6.82 mm, respectively. These frequencies represent a compromise between achieving sufficient penetration of the induced eddy currents to interact with the investigated subsurface defects and maintaining an adequate magnetic field intensity for defect detection.

The numerical investigation exclusively considers subsurface defects. This choice was motivated by the objective of evaluating the effectiveness of the proposed ferrite-core probe under the operating conditions where conventional air-core ECT probes generally exhibit the largest reduction in inspection capability owing to the combined effects of eddy current attenuation and reduced magnetic field intensity.

### 2.3. Obtained Numerical Results

The numerical investigation was first carried out considering a conductive specimen without defect in order to isolate the electromagnetic contribution introduced by the ferrite cores from the perturbations generated by the presence of a defect. The analysis focused on the *z*-component of the magnetic flux density ($B_{z}$) generated by the excitation system and on the corresponding eddy current density induced within the conductive specimen, since these quantities directly determine the inspection capability of the proposed probe.

**Figure 4:**
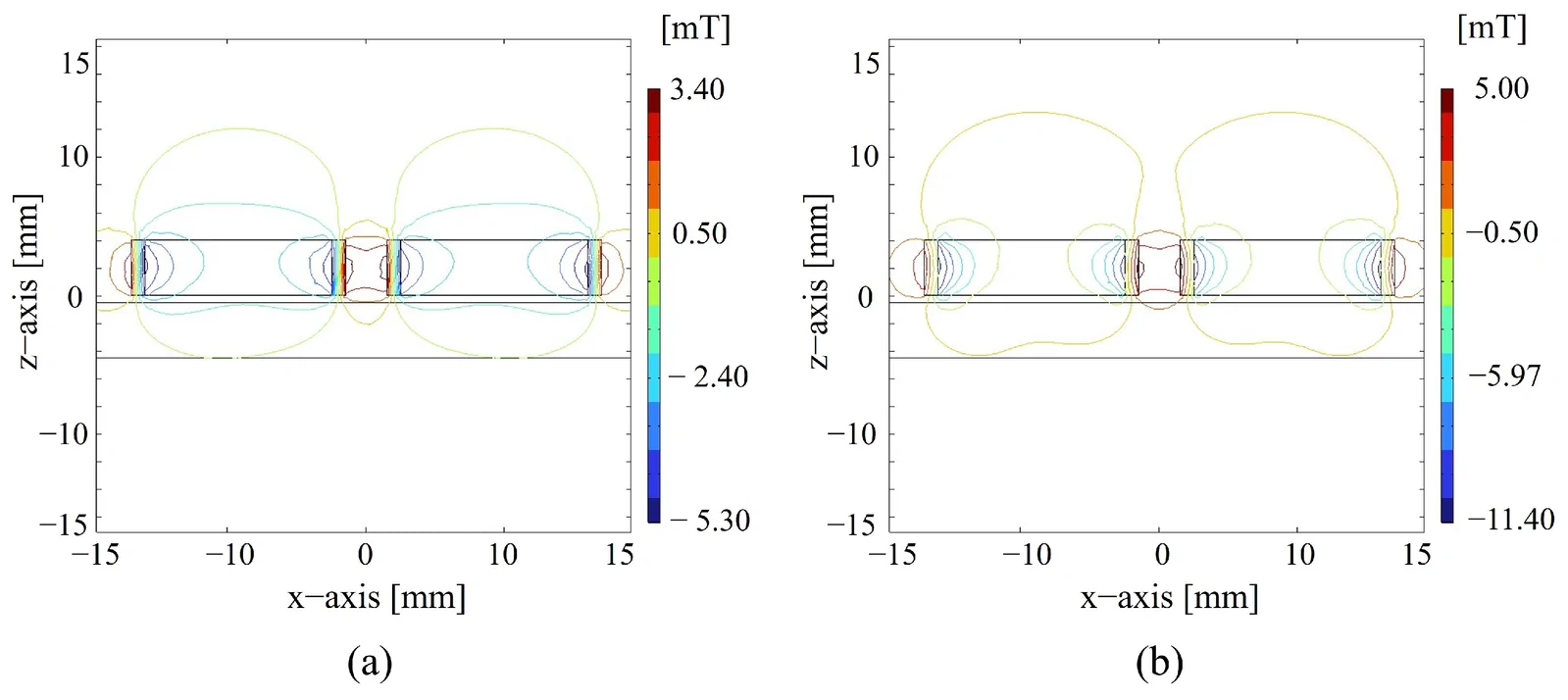
Distribution of the magnetic flux density in z direction generated by the proposed probe: (**a**) air-core configuration; (**b**) ferrite-core configuration.

Figure 4 compares the distributions of the *z*-component of the magnetic flux density ($B_{z}$) obtained for the air-core and ferrite-core probe configurations. In the conventional air-core configuration (Figure 4a), a significant portion of the magnetic flux spreads through the surrounding air before reaching the conductive specimen. Consequently, only part of the generated magnetic field effectively contributes to the induction of eddy currents within the inspected material. By contrast, the ferrite-core configuration (Figure 4b) concentrates the magnetic flux below the excitation coils due to the higher magnetic permeability of the ferrite inserts. This behavior significantly reduces the magnetic flux leakage into the surrounding air while increasing the magnitude of the *z*-component of the magnetic flux density within the inspected region. In particular, $B_{z}$ values ranged from −5.30 to 3.40 mT for the air-core configuration and from −11.40 to 5.00 mT for the ferrite-core configuration, corresponding to an increase of approximately 47%.

**Figure 5:**
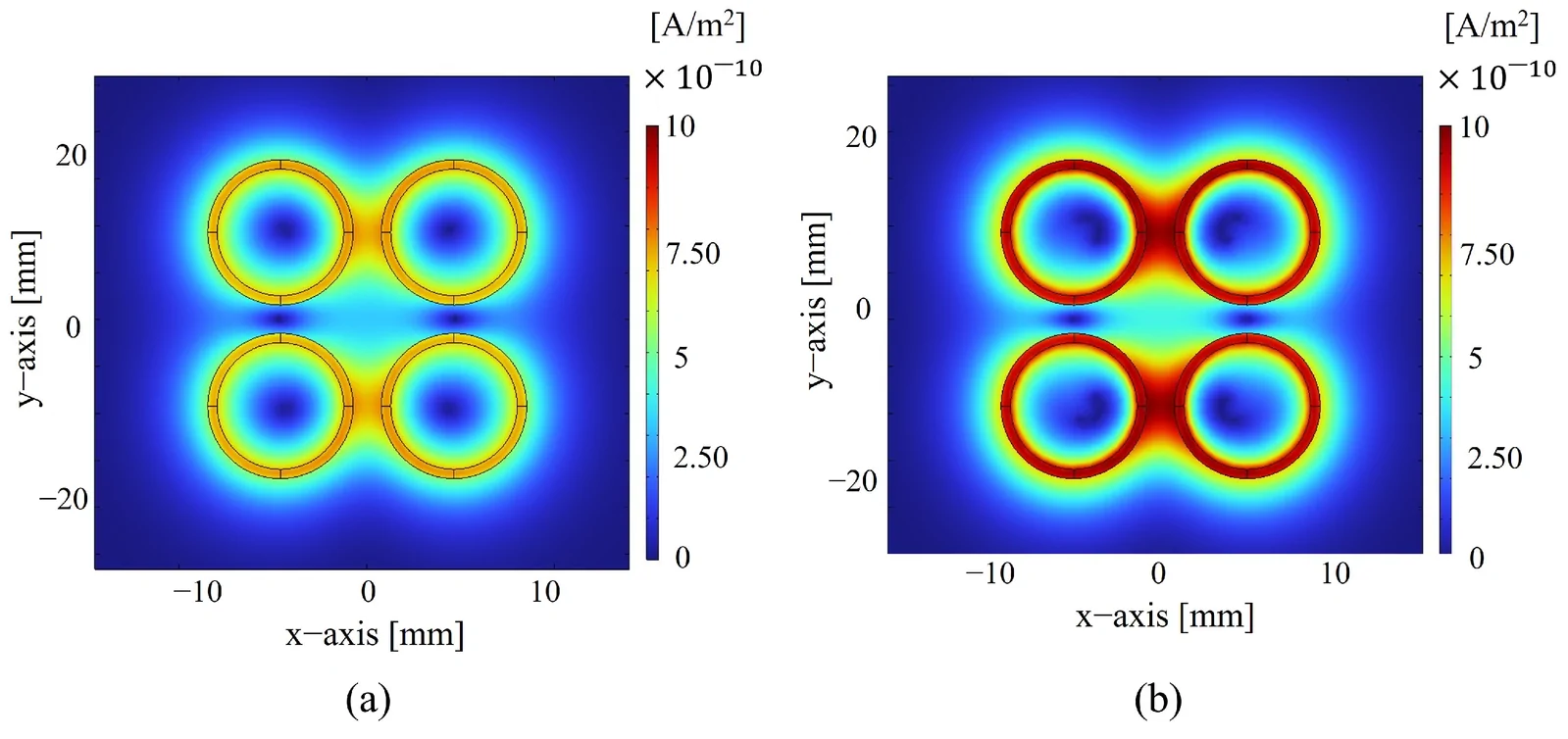
Surface distribution of the induced eddy current density: (**a**) air-core configuration; (**b**) ferrite-core configuration.

The increase in the magnetic field intensity directly affects the eddy current distribution induced within the conductive specimen. Figure 5 reports the corresponding surface distributions of the induced eddy current density for the two probe configurations. Although the ferrite inserts do not modify the overall eddy current paths generated by the adopted REC excitation strategy, they significantly increase the magnitude of the induced currents beneath the excitation coils. In particular, the maximum eddy current density increases from approximately $5\times 10^{-10}$ A/m^2^ for the air-core configuration to about $8\times 10^{-10}$ A/m^2^ for the ferrite-core configuration, corresponding to an increase of approximately 60%. This behavior results from the stronger magnetic coupling established between the excitation system and the conductive specimen, leading to a more intense electromagnetic interaction within the inspected region.

Overall, the numerical analysis demonstrates that the ferrite-core configuration enhances the electromagnetic coupling between the excitation system and the conductive specimen without modifying the operating principle of the proposed REC probe. The simultaneous increase in the magnetic field intensity and the induced eddy current density is therefore expected to produce a stronger reaction magnetic field in the presence of a defect, resulting in an enhanced inspection capability. Based on these observations, the following analysis quantitatively evaluates the performance improvement introduced by the ferrite cores in the presence of subsurface defects to be inspected.

To reproduce the experimental inspection procedure, the numerical analysis was performed by virtually scanning the probe over a 40×40 mm^2^ area centered on the investigated defect, with a spatial step of 1 mm. At each scanning position, the magnetic field was evaluated at a numerical sensing point located at the center of the probe, corresponding to the position of the pick-up sensor shown in Figure 1. Consistently with the adopted probe reference system, only the *z*-component of the magnetic field was considered throughout the numerical investigation. This choice is motivated by the results presented in [24], where the *z*-component was demonstrated to provide the highest sensitivity to crack-induced magnetic field perturbations among the three measured components.

**Figure 6:**
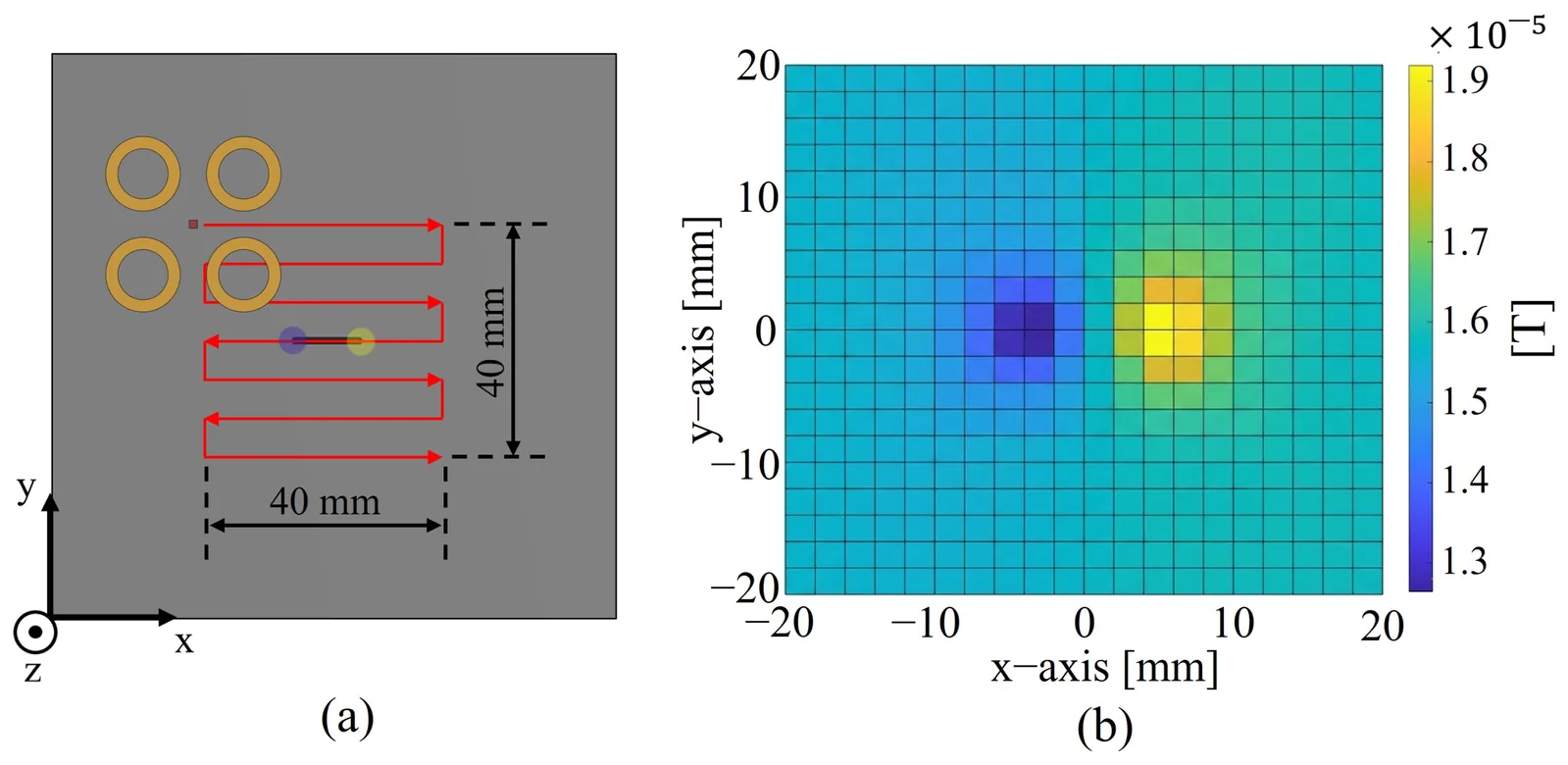
Numerical inspection procedure: (**a**) probe, defect, and scanning area; (**b**) representative $B_{z}$ map over the scanned area.

The adopted numerical scanning procedure and the corresponding evaluation of the probe response are schematically illustrated in Figure 6. Figure 6a shows the probe position with respect to the investigated defect and the scanning area, whereas Figure 6b reports a representative map of the resulting $B_{z}$ distribution over the scanned area. The maximum and minimum values of the magnetic field, denoted as $B_{z,\max}$ and $B_{z,\min}$, respectively, are highlighted in the map, with the yellow region corresponding to $B_{z,\max}$ and the blue region to $B_{z,\min}$.

To quantitatively compare the air-core and ferrite-core configurations, the numerical performance was evaluated through the variation of the magnetic field over each simulated scanning map. Accordingly, the numerical figure of merit, ΔB, was defined as

(3) $$\Delta B=B_{z,\max}-B_{z,\min},$$

where $B_{z,\max}$ and $B_{z,\min}$ are respectively the maximum and minimum magnetic field values extracted from the simulated scanning map.

The numerical analysis was repeated for the different investigated crack orientations, namely $\varphi =0^{\circ }$, $45^{\circ }$, $60^{\circ }$, $90^{\circ }$, and $135^{\circ }$. For each value of φ, the complete rotation of the eddy current distribution was considered and the maximum value of ΔB was extracted. No significant variations in the maximum ΔB were observed among the different crack orientations, since, for each value of φ, the maximum response was consistently obtained when the eddy current distribution was approximately orthogonal to the main crack dimension (i.e., $\left|\theta -\varphi \right|=90^{\circ }$). This result confirms that the REC procedure allows comparable maximum defect responses to be obtained independently of the crack orientation. Therefore, for the sake of conciseness, Table 3 reports the maximum ΔB values obtained under the orthogonality condition for each investigated defect with an orientation angle $\varphi =0^{\circ }$.

The improvement introduced by the ferrite cores was quantified through the relative variation of the figure of merit with respect to the air-core configuration, according to

(4) $$\eta =\frac{\Delta B_{\mathrm{Ferrite}}-\Delta B_{\mathrm{Air}}}{\Delta B_{\mathrm{Air}}}\times 100.$$

Table 3 summarizes the numerical comparison between the air-core and ferrite-core probe configurations in terms of the adopted figure of merit and the corresponding improvement.

As can be observed, the ferrite-core configuration systematically outperforms the air-core probe for all the investigated defects. Moreover, the percentage improvement remains nearly constant for both specimens, ranging between approximately 28% and 39%.

## 3. Experimental Set-Up and Case Studies

**Figure 7:**
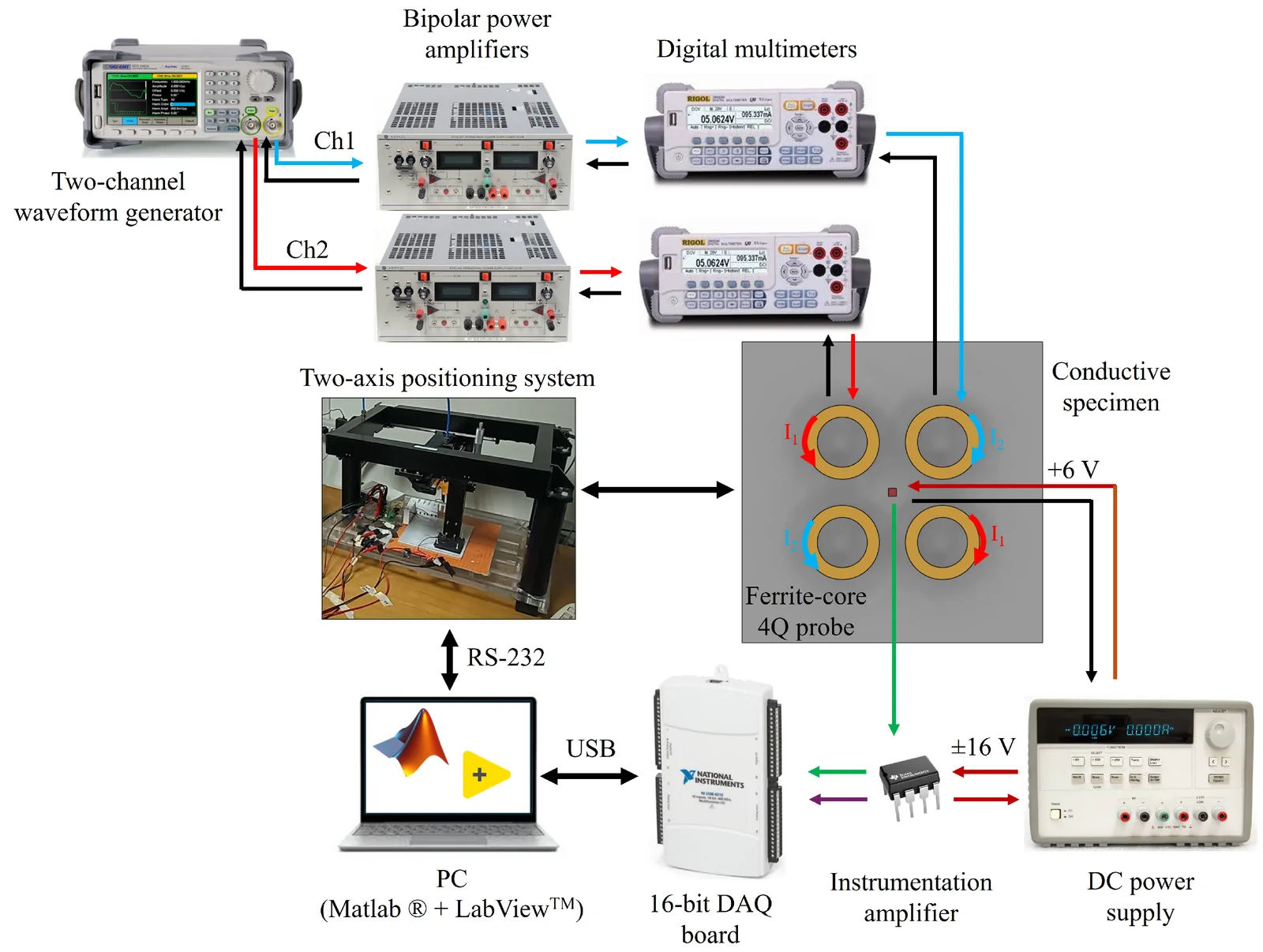
Schematic representation of the adopted measurement set-up.

The experimental validation was carried out by means of the measurement set-up schematically illustrated in Figure 7.

The excitation system is based on a two-channel arbitrary waveform generator (SDG1032X, Siglent Technologies Co., Shenzhen, China), responsible for generating the excitation currents $I_{1}$ and $I_{2}$ supplied to the two excitation coil pairs (i.e., $C_{1}$–$C_{3}$ and $C_{2}$–$C_{4}$) composing the probe. Since the current delivered by the waveform generator is not sufficient to directly drive the excitation coils, two bipolar power amplifiers (BOP 20-20M, Kepco Inc., New York, NY, USA) were employed. The current flowing in each excitation channel was continuously monitored by two digital multimeters (DM3058, Rigol Technologies Co., Suzhou, China) to guarantee the prescribed excitation conditions throughout the entire measurement procedure.

To ensure consistency between the numerical and experimental investigations, both the excitation current amplitudes and frequencies were set to the same values adopted in the finite element analysis. For each defect orientation, the base excitation current was set to $I_{b}=300$ mA, while $I_{1}$ and $I_{2}$ were varied according to (1) and (2) to rotate the resultant eddy current distribution over a complete rotation with an angular resolution of $5^{\circ }$.

The magnetic field was measured using a tunnel magnetoresistance (TMR) sensor (TMR2905, MultiDimension Technology Co., Zhangjiagang, China), which was selected for its high sensitivity and compact size. The TMR sensor was positioned at the center of the four excitation coils with its sensitive axis aligned with the z-direction (consistently with the numerical investigation) and was supplied by a stabilized DC power supply (E3631A, Agilent Technologies Inc., Santa Clara, CA, USA) with a supply voltage of +6 V. Its differential output voltage was conditioned by an instrumentation amplifier (INA217, Texas Instruments, Dallas, TX, USA), powered at ±16 V, before being digitized by a 16-bit data acquisition board (USB-6212, National Instruments, Austin, TX, USA).

The developed ferrite-core 4Q probe was manufactured according to the geometrical parameters reported in Table 1. For both specimen configurations, the sampling frequency was set to 100 times the corresponding excitation frequency, while the acquisition window included 10 excitation periods.

The entire inspection procedure was managed by a dedicated LabVIEW application, which simultaneously controlled the measurement instrumentation and the automated scanning platform. The probe was mounted on a computer-controlled two-axis positioning system, which allowed automatic inspection of the specimen surface while maintaining a constant lift-off of 0.5 mm. For each experiment, the probe scanned a 40×40 mm^2^ area centered on the investigated defect with a spatial step of 1 mm and the specimen was physically rotated in order to set the different defect orientations.

Once the acquisition procedure was completed, the recorded signals were processed in MATLAB R2024b by means of a Fast Fourier Transform (FFT). For each scanning position, the magnitude of the spectral component corresponding to the excitation frequency was extracted from the acquired TMR voltage signal and assigned to the corresponding scanning point. Repeating this procedure over the entire inspection area allowed the reconstruction of the voltage amplitude map.

In agreement with the numerical analysis, the experimental performance was quantified through the variation of the voltage amplitude over each scanning map. Accordingly, the following figure of merit was adopted

(5) $$\Delta V=V_{\max}-V_{\min},$$

where $V_{\max}$ and $V_{\min}$ denote the maximum and minimum voltage values measured over the scanned area, respectively. Larger values of ΔV correspond to a stronger perturbation of the magnetic field induced by the defect and, consequently, to a higher defect detectability of the proposed eddy current probe.

Consistently with the numerical procedure described in Section 2.3, for each investigated defect orientation φ, ΔV was evaluated over the complete rotation of the eddy current distribution, and its maximum value was considered for the performance evaluation. In all cases, the maximum response was obtained when the eddy current distribution was approximately orthogonal to the main crack dimension (i.e., $\left|\theta -\varphi \right|=90^{\circ }$).

**Figure 8:**
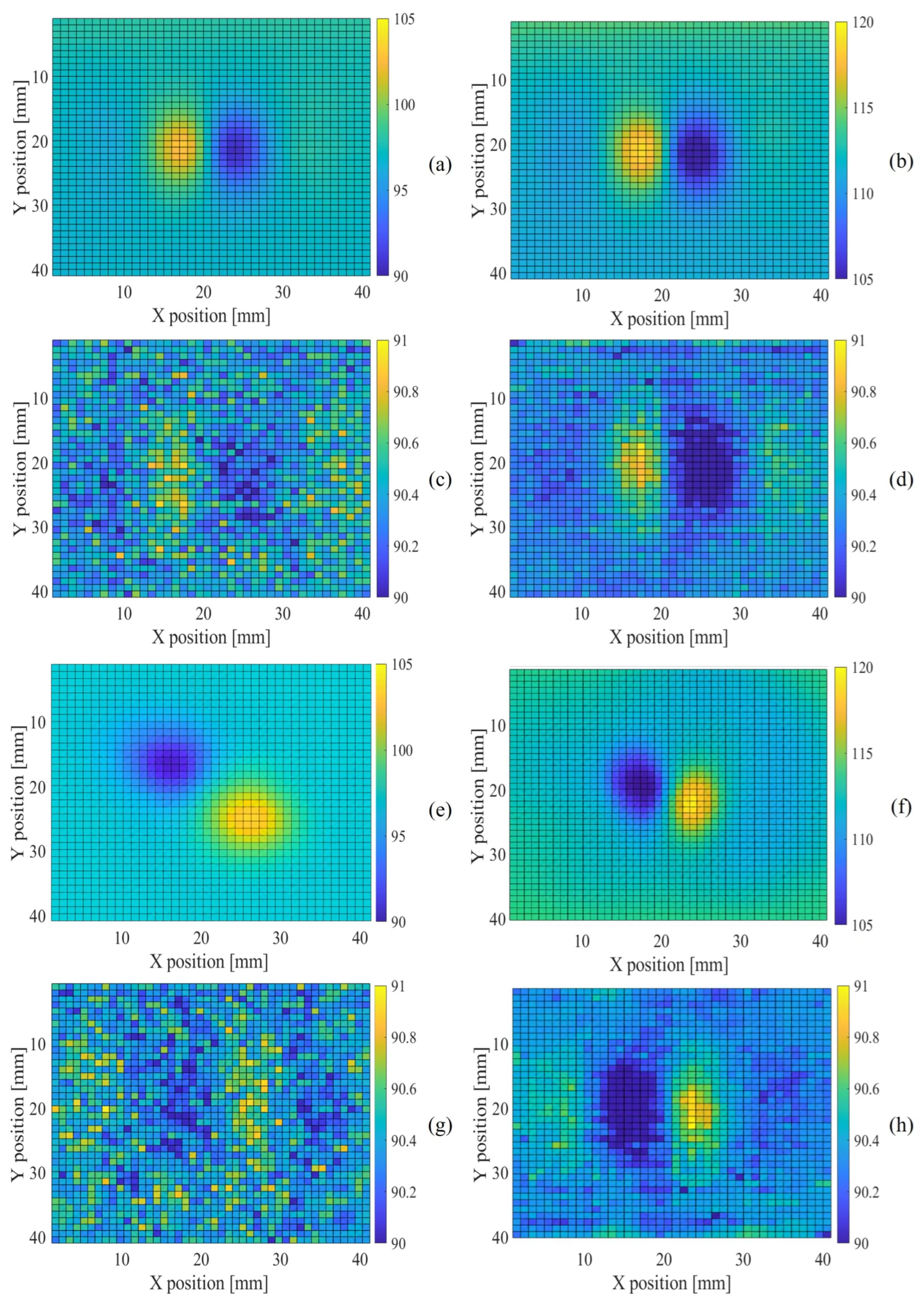
Experimental voltage amplitude maps obtained for defects D1 and D10 using the air-core and ferrite-core probe configurations at two representative defect orientations: (**a**) D1, air-core, $\varphi =0^{\circ }$; (**b**) D1, ferrite-core, $\varphi =0^{\circ }$; (**c**) D10, air-core, $\varphi =0^{\circ }$; (**d**) D10, ferrite-core, $\varphi =0^{\circ }$; (**e**) D1, air-core, $\varphi =135^{\circ }$; (**f**) D1, ferrite-core, $\varphi =135^{\circ }$; (**g**) D10, air-core, $\varphi =135^{\circ }$; (**h**) D10, ferrite-core, $\varphi =135^{\circ }$.

Representative examples of the obtained voltage amplitude maps are shown in Figure 8 for defects D1 and D10 at two different defect orientations, namely $\varphi =0^{\circ }$ and $\varphi =135^{\circ }$. For each defect orientation, Figure 8 reports the maps characterized by the maximum ΔV obtained through the REC procedure.

The conductive specimens and the investigated crack geometries match the case studies introduced in Section 2.2. Consequently, the experimental campaign was specifically designed to validate the numerical predictions by comparing the ferrite-core and the corresponding air-core probe configurations under identical operating conditions.

## 4. Experimental Results and Discussion

The experimental campaign was carried out to validate the numerical investigation presented in Section 2 through a direct comparison between the ferrite-core and air-core probe configurations. To this end, two experimental campaigns were performed using the same 4Q probe. In the first campaign, the probe operated in the conventional air-core configuration, whereas in the second one ferrite cores were inserted inside the four excitation coils. All the remaining experimental conditions were kept unchanged to isolate the contribution introduced by the ferrite cores.

Figure 8 reports representative experimental voltage amplitude maps obtained for defects D1 and D10 at two different defect orientations. In particular, Figure 8a–d show the results obtained for $\varphi =0^{\circ }$, with panels (a) and (b) referring to defect D1 for the air-core and ferrite-core configurations, respectively, and panels (c) and (d) referring to defect D10. Similarly, Figure 8e–h report the corresponding results obtained after rotating the specimen to obtain a defect orientation of $\varphi =135^{\circ }$.

A comparison between the two investigated orientations shows that, for both D1 and D10, the maximum values of the figure of merit ΔV obtained through the REC procedure remain approximately unchanged when the defect orientation is varied from $\varphi =0^{\circ }$ to $\varphi =135^{\circ }$. This experimental evidence confirms that the REC procedure can compensate for changes in defect orientation by properly rotating the induced eddy current distribution, thus providing comparable maximum responses for differently oriented defects.

Considering the probe configurations, for the shallowest defect D1, both the air-core and ferrite-core probes successfully identify the presence and position of the defect for both orientations. Nevertheless, the ferrite-core configuration provides a larger voltage variation over the scanned area, resulting in enhanced defect detectability. A different scenario is observed for the deepest investigated defect D10. In this case, for both $\varphi =0^{\circ }$ and $\varphi =135^{\circ }$, measurement noise masks the defect-induced signal when the air-core configuration is used. Conversely, the ferrite-core configuration still provides a distinguishable voltage variation in the defect region. These results confirm that the magnetic flux concentration provided by the ferrite cores becomes particularly beneficial under the low-frequency operating conditions required for deep subsurface defect detection.

Since comparable maximum ΔV values were obtained for the different investigated defect orientations, for the sake of conciseness, the quantitative comparison reported in Table 4 refers to the results obtained for $\varphi =0^{\circ }$.

In agreement with the numerical investigation, the improvement introduced by the ferrite cores was quantified through the relative variation of the experimental figure of merit, ΔV, with respect to the air-core configuration. Accordingly, the experimental relative improvement, $\eta_{\exp}$, was defined as

(6) $$\eta_{exp}=\frac{\Delta V_{\mathrm{Ferrite}}-\Delta V_{\mathrm{Air}}}{\Delta V_{\mathrm{Air}}}\times 100.$$

The experimental results reported in Table 4 consistently confirm the effectiveness of the proposed ferrite-core configuration. For all the investigated defects, the ferrite-core configuration provides larger values of the adopted figure of merit than the corresponding air-core configuration, demonstrating that the increase in magnetic flux concentration effectively translates into an enhanced defect detectability.

For the specimen with 4 mm of thickness, inspected at an excitation frequency of 500 Hz, the improvement introduced by the ferrite cores remains nearly constant, ranging from approximately 22% to 26%. By contrast, a different trend is observed for the 10 mm specimen inspected at 200 Hz. In this case, the improvement progressively increases as the inspection conditions become more challenging, reaching values exceeding 100% for defect D9. Furthermore, defect D10 could only be detected using the ferrite-core configuration, whereas no measurable signal variation due to the defect was obtained with the corresponding air-core probe.

Although the experimental improvements differ quantitatively from those predicted by the numerical model, both investigations exhibit the same overall behavior and lead to consistent conclusions. The observed discrepancies can be reasonably attributed to factors excluded from the finite element model, including the metrological characteristics of the measurement chain, the actual behavior and noise of the magnetic sensor, manufacturing tolerances of the probe, and experimental uncertainties, whose influence becomes increasingly relevant for deeper defects as the signal response related to the defect progressively decreases.

Overall, the experimental investigation demonstrates that the proposed ferrite-core configuration provides progressively larger benefits as the inspection conditions become more demanding. This behavior is particularly relevant from an application perspective, as the largest improvements are achieved precisely under the operating conditions where conventional REC systems typically experience the greatest reduction in inspection capability, namely low-frequency excitation and deep subsurface inspections.

Finally, Table 5 compares the proposed probe with representative ECT approaches for subsurface defect detection considering the dependence on defect orientation. Previous ferrite-based solutions have demonstrated effective subsurface inspection, but without providing orientation-independent detection. Conversely, existing ferrite-core REC probes provide orientation-independent inspection, but their experimental validation has been limited to superficial cracks and relatively high excitation frequencies [8,15,23,25]. The present work combines these two capabilities, experimentally demonstrating the detection of arbitrarily oriented defects buried up to 6 mm under low-frequency excitation. It should be noted that the reported detection depths cannot be directly interpreted as a ranking of probe performance, since the compared studies differ in materials, defect geometries, excitation conditions, and sensing strategies.

## 5. Conclusions

This paper proposes a novel ferrite-core Eddy Current Testing (ECT) probe designed to combine the orientation-independent inspection capability of the Rotating Eddy Current (REC) excitation strategy with the magnetic flux concentration provided by the ferrite cores, thereby enhancing the detectability of deep, arbitrarily oriented subsurface defects.

The proposed solution was first investigated through finite element analysis and subsequently validated through an experimental campaign under the same operating conditions. Both numerical and experimental results consistently demonstrated that the ferrite-core configuration enhances the inspection capability of the proposed probe. While improvements of approximately 22% were obtained for shallow subsurface defects, the benefits progressively increased as the inspection conditions became more challenging, exceeding 100% for the deepest detectable defects. Furthermore, the ferrite-core probe successfully detected discontinuities buried up to 6 mm below the inspected surface, whereas the corresponding air-core configuration failed under the same operating conditions.

Overall, the obtained results demonstrate that ferrite-core integration represents an effective solution for extending the applicability of REC probes to low-frequency inspection of deep subsurface defects while preserving their capability to inspect defects regardless of their orientation.

Future work will focus on the optimization of the ferrite-core geometry and magnetic material, as well as on extending the proposed system to multi-frequency inspection strategies and quantitative defect characterization.

## Figures and Tables

**Table 1:** Main geometrical parameters of the proposed ferrite-core Four-Quadrant probe.

Parameter	Value
$d_{ext}$	15.5 mm
$d_{inn}$	13.5 mm
$d_{x}$	0.5 mm
$d_{y}$	0.5 mm
$h_{c}$	4 mm
$l_{0}$	0.5 mm
Number of turns (*N*)	120
Ferrite relative permeability ($\mu_{r}$)	5

**Table 2:** Main geometrical characteristics of the investigated cracks.

Specimen	Code	*l*	*t*	*h*	*d*	φ
Thickness [mm]		[mm]	[mm]	[mm]	[mm]	[°]
4	D1	5	0.1	3	1	0, 45, 60, 90, 135
	D2			2	2	
	D3			1.5	2.5	
	D4			1	3	
10	D5	5	0.2	9	1	
	D6			8	2	
	D7			7	3	
	D8			6	4	
	D9			5	5	
	D10			4	6	

**Table 3:** Numerical comparison between the air-core and ferrite-core probe configurations for a defect orientation of $\varphi =0^{\circ }$.

Code	$\Delta B_{\mathrm{Air}}$ [mT]	$\Delta B_{\mathrm{Ferrite}}$ [mT]	η [%]
D1	$4.44\times 10^{-2}$	$5.79\times 10^{-2}$	30.52
D2	$1.55\times 10^{-2}$	$2.02\times 10^{-2}$	29.89
D3	$8.16\times 10^{-3}$	$1.06\times 10^{-2}$	29.37
D4	$3.52\times 10^{-3}$	$4.51\times 10^{-3}$	28.22
D5	$2.94\times 10^{-2}$	$3.98\times 10^{-2}$	35.60
D6	$1.44\times 10^{-2}$	$1.94\times 10^{-2}$	34.99
D7	$7.61\times 10^{-3}$	$1.02\times 10^{-2}$	34.37
D8	$4.00\times 10^{-3}$	$5.46\times 10^{-3}$	36.49
D9	$2.10\times 10^{-3}$	$2.91\times 10^{-3}$	38.79
D10	$1.05\times 10^{-3}$	$1.46\times 10^{-3}$	39.13

**Table 4:** Experimental comparison between the air-core and ferrite-core probe configurations for a defect orientation of $\varphi =0^{\circ }$.

Code	$\Delta V_{\mathrm{Air}}$ [mV]	$\Delta V_{\mathrm{Ferrite}}$ [mV]	$\eta_{\exp}$ [%]
D1	12.40	15.42	24.32
D2	4.73	5.96	25.96
D3	2.57	3.23	25.81
D4	1.26	1.54	22.21
D5	7.85	10.11	28.80
D6	4.30	5.77	34.07
D7	2.32	3.65	57.14
D8	1.04	1.85	77.59
D9	0.51	1.07	109.65
D10	N.D.	0.83	–

**Table 5:** Comparison of representative ECT approaches for subsurface defect detection considering the dependence on defect orientation.

Reference	Approach	Frequency	Investigated Defects	Max. Defect Depth	Dependence on Defect Orientation
Liu et al. [15]	Bidirectional ECT + ferrite shielding	1 kHz	Subsurface notches/holes	7 mm	Yes
Xie et al. [8]	Differential PECT + ferrite pot	Pulsed	Subsurface cracks	8 mm	Yes
Trung et al. [25]	REC + ferrite cores	10 kHz	Superficial cracks	–	No
Trung et al. [23]	REC + ferrite cores	10 kHz	Superficial cracks	–	No
Present work	REC + ferrite cores	200–500 Hz	Subsurface cracks	6 mm	No

## Data Availability

The original contributions presented in this study are included in the article. Further inquiries can be directed to the corresponding author.
